# Dietary Puerarin Supplementation Alleviates Oxidative Stress in the Small Intestines of Diquat-Challenged Piglets

**DOI:** 10.3390/ani10040631

**Published:** 2020-04-07

**Authors:** Meng Li, Daixu Yuan, Yanhong Liu, Hui Jin, Bie Tan

**Affiliations:** 1 Laboratory of Animal Nutritional Physiology and Metabolic Process, Key Laboratory of Agro-ecological Processes in Subtropical Region, National Engineering Laboratory for Pollution Control and Waste Utilization in Livestock and Poultry Production, Institute of Subtropical Agriculture, Chinese Academy of Sciences, Changsha 410125, China; limeng183@mails.ucas.ac.cn (M.L.); jinh7674@163.com (H.J.); 2 University of Chinese Academy of Sciences, Beijing 100008, China; 3 Department of Medicine, Jishou University, Jishou 416000, China; yuandaixiu123@126.com; 4 Department of Animal Science, University of California, Davis, CA 95616, USA; yahliu@ucdavis.edu; 5 College of Animal Science and Technology, Hunan Agricultural University, Changsha 410128, China

**Keywords:** puerarin, diquat, piglets, oxidative stress, Nrf2 pathway

## Abstract

**Simple Summary:**

The oxidant stress which piglets suffer from during the weaning period has caused huge losses to the pig farm industry. It is important for scientists to find an effective way to alleviate the oxidant stress in weaned piglets. This study was designed to test the hypothesis that dietary puerarin supplementation alleviates oxidative stress in the small intestine of diquat-challenged piglets. Interestingly, dietary puerarin supplementation improved intestinal morphology, cell proliferation, barrier function, and increased Nrf2 and its downstream enzymes in diquat-challenged piglets, which shows that puerarin has potent protective effects against diquat-induced oxidative stress. These findings will be very beneficial to the pig industry, especially to the development of antibiotic-free diets, new anti-inflammatory drugs and the application of puerarin in piglets.

**Abstract:**

This study was conducted to demonstrate that dietary puerarin supplementation alleviates oxidative stress in the small intestine of diquat-challenged piglets. The results showed that puerarin administration markedly alleviated diquat-induced intestinal injury, which was indicated by the improvement of intestinal morphology, cell proliferation and barrier function. One of the potential mechanisms responsible for this was the decrease in oxidative stress, as evidenced by the increase in activities of superoxide dismutase (SOD), glutathione peroxidase (GSH-Px), and total antioxidant capacity (T-AOC) in the small intestine. Puerarin increased the protein expression levels of NF-E2-related factor 2 (Nrf2) and its downstream enzymes, including heme oxygenase 1 (HO-1), glutamate–cysteine ligase catalytic and its modifier subunit (GCLc and GCLm) in the jejunal mucosa of diquat-induced piglets. Puerarin administration improved intestinal morphology, cell proliferation, and barrier function, and increased Nrf2 and its downstream enzymes. These findings indicate that the dietary supplementation of puerarin attenuates the oxidative stress involving Nrf2 signaling pathways in diquat-challenged piglets.

## 1. Introduction

Piglets are prone to oxidative balance disruption and oxidative injury during the weaning period. The previous study demonstrated that weaning is associated with oxidative injury of the lipid, protein, and DNA, as well as declines in the activities of the intestinal antioxidant enzymes under the p65 and NF-E2-related factor 2 (Nrf2) signals [1]. Under normal physiological conditions, the production of oxidants and antioxidants is balanced in biological systems [2]. Oxidative stress occurs when antioxidant systems cannot neutralize the excess reactive oxygen species (ROS) produced in cells and tissues [3]. As the products of mitochondrial metabolism of the eukaryotic cell, ROS are capable of initiating oxidation and play an important role in maintaining cell homeostasis and regulating signal transduction, gene expression, and enzyme activation at low levels [4,5].

The supplementation of exogenous antioxidants may help restore the pro-oxidative–antioxidative balance [6]. Puerarin, a natural isoflavone compound, has been reported to have strong antioxidant activities and exert a wide range of beneficial effects [7,8,9]. Puerarin can scavenge free radicals, inhibit pro-inflammatory cytokines, decrease inflammatory genes, up-regulate antioxidant enzymes, modulate transcription factors, and enhance the Nrf2 signaling pathway [10,11]. It has been reported that puerarin effectively inhibited inflammation in the kidney induced by both lipopolysaccharide and AGEs in mouse mesangial cells [12,13]. Puerarin also exerted renal protective effects in diabetic rats and renovascular hypertensive rats [14,15]. However, the protective effects of puerarin on oxidative stress in the intestines of weanling piglets have rarely been reported. 

Therefore, the current study was designed to explore the protective roles of puerarin in piglets suffering from oxidative stress. We hypothesized that puerarin would exert protective effects on oxidative stress in the intestines of diquat-challenged piglets, which may involve Nrf2 signaling pathways.

## 2. Materials and Methods

### 2.1. Animals and Experimental Design

The animal experiments were approved by the Institutional Animal Care and Use Committee of the Institute of Subtropical Agriculture, Chinese Academy of Sciences (2013020).

A total of 48 healthy piglets with body weight (BW) 7.26 ± 0.51 kg weaned at 21 days were randomly assigned to receive one of three treatments, with eight replicate pens per treatment, and two piglets per pen. The three treatments included a basal diet, a basal diet + diquat, and a 0.1‰ puerarin diet + diquat. (The puerarin dose was based on the growth performance of piglets in the preliminary experiment.) Diquat was purchased from Sigma-Aldrich (St. Louis, MO, USA). The basal diets were designed to meet the nutrient requirements for weaned piglets, as shown in Table 1. The piglets were housed in a room with hard plastic-slatted flooring. All piglets had free access to drinking water. 

After an adaptation period of seven days, the piglets were fed their respective diets three times per day for a 14-day experimental period. On day 7 after the initiation of treatment, the piglets on the basal diet + diquat and 0.1‰ puerarin + diquat treatments received an intraperitoneal injection of diquat at 8 mg/kg BW (a dose of 8 mg/kg BW was used according to the results reported by Yin et al) [16], while the piglets on the control treatment received the same volume of sterilized saline. On day 14, 24 piglets (one pig per pen) were randomly selected and slaughtered. The intestinal samples were collected from the center of the jejunum and ileum and were stored at −80 °C or fixed in a formaldehyde solution. Weight gain and feed intake of each treatment were calculated on the basis of the average value of each pen.

### 2.2. Intestinal Morphology

The fixed samples of the jejunum and ileum in the formaldehyde solution were embedded in paraffin. Using a microtome, cross sections of the samples were cut to an approximate thickness of 5 μm. Then, hematoxylin–eosin staining was performed with the procedures of dehydration, embedding, sectioning, and staining. Villous height and crypt depth were measured with computer-assisted microscopy [17] (Micrometrics TM; Nikon ECLIPSE E200, Tokyo, Japan).

### 2.3. Intestinal Mucosal Protein, DNA and RNA 

The concentrations of protein, DNA and RNA in the jejunal and ileal mucosa were measured by a BCA assay kit, fluorometric assay and spectrophotometry, respectively, according to the methods of Liu et al [18].

### 2.4. Immunohistochemical Analysis

The expressions of the proliferating cell nuclear antigen (PCNA) in the jejunal and ileal mucosa were determined using immunohistochemical analysis in accordance with the methods of Wang et al. (2015). The anti-PCNA (1:200; Wuhan Boster Biological Technology Co., Ltd., Wuhan, China) and an SV mouse or rabbit hypersensitivity 2-step immunohistochemical kit (Boster Biological Technology) were used. The stained sections were reviewed and scored independently by two investigators using a microscope (Olympus, Tokyo, Japan). The PCNA labeling index was expressed as the ratio of cells that were positively stained for PCNA to all epithelial cells in at least five areas that were randomly selected for counting at less than 200-fold magnification. All data were expressed as the relative values to those of the control piglets. 

### 2.5. Real-Time Quantitative Reverse Transcriptase PCR

The expressions of E-cadherin, occludin and zonula occludens (ZO-1) in the jejunal and ileal mucosa were determined by real-time quantitative reverse transcriptase PCR (real-time qRT-PCR). Primers were designed with Primer 5.0 (PREMIER Biosoft International, Palo Atlo, CA, USA) according to the gene sequence of the pig, to produce an amplification product that was shown in the previous study [19]. Beta-actin was used as a housekeeping gene to normalize target gene transcript levels [20]. The comparative threshold cycle (Ct) value method was employed to the quantitative expression levels of the target genes relative to those of β-Actin. Data are expressed as the relative values to those of the control piglets [19].

### 2.6. Antioxidative Capacity of Intestinal Mucosa

Jejunal and ileal mucosal concentrations of superoxide dismutase (SOD), glutathione peroxidase (GSH-Px), catalase (CAT), malondialdehyde (MDA), total antioxidant capacity (T-AOC), and glutathione (GSH) were measured using their corresponding assay kits (Nanjing Jiancheng, Nanjing, China) according to manufacturer instructions. SOD, CAT, and GSH-Px were analyzed by the xanthine–oxidase-xanthine reaction method, the CAT-H_2_O_2_ reaction method, and the reduced glutathione method, respectively. MDA capacity was assayed by the 2-thiobarbituric acid method and T-AOC was detected by the ferric-reducing antioxidant power reaction method. All samples were measured by UV-visible spectrophotometry (UV-2450, Shimadzu, Kyoto, Japan).

### 2.7. Western Blot Analysis

Jejunal mucosal samples were homogenized and protein concentrations were measured using the bicinchoninic acid assay method with BSA as the standard (Beyotime Institute of Biotechnology, Shanghai, China). All samples were adjusted to an equal concentration (50µg protein). The antibodies used in this study were as follows: Nrf2 antibody (1:500, Santa Cruz Biotechnology), heme oxygenase 1 (HO-1) antibody (1:500, Bioss Inc.), NAD(P)H:quinone oxidoreductase 1 (NQO-1) antibody (1:10000, Invitrogen), glutamate–cysteine ligase catalytic subunit (GCLc) antibody (1:1000, LifeSpan BioSciences), glutamate–cysteine ligase modifier (GCLm) antibody (1:1000, LifeSpan BioSciences) and β-actin (1:400; Santa Cruz Biotechnology). The expression ratio of the target proteins was normalized against β-actin, and the data are expressed relative to the values of the piglets on the basal diet treatment.

### 2.8. Statistical Analysis

All data were subjected to analysis of variance (ANOVA) using SPSS 17.0 software (SPSS, Inc., Chicago, IL, USA). The differences among the treatments were evaluated using Tukey’s test. Probability values < 0.05 were taken to indicate statistical significance.

## 3. Results

### 3.1. Growth Performance 

The results of the growth performance are shown in Table 2. The injection of diquat significantly decreased (*p* < 0.05) the final BW, average daily weight gain (ADG), average daily feed intake (ADFI) and gain:feed (G:F) ratio in the piglets. However, the growth performance of the piglets on the puerarin diet + diquat treatment showed no difference compared to those on the basal diet and basal diet + diquat treatments (*p* > 0.05). 

### 3.2. Jejunal and Ileal Morphology

In the jejunum, diquat challenge reduced (*p* < 0.05) villous height and the ratio of villous height to crypt depth. The ratio of villous height to crypt depth in the piglets on the puerarin diet + diquat treatment was greater than those on the basal diet + puerarin treatment (*p* < 0.05), as shown in Table 3. 

In the ileum, the ratio of villous height to crypt depth in the piglets on the basal diet + diquat treatment was decreased in comparison to those on the basal diet treatment (*p* < 0.05). However, no differences were observed in villous height, crypt depth and the ratio of villous height to crypt depth between the piglets on the basal diet + diquat treatment and those on the puerarin diet + diquat treatment (*p* > 0.05), as shown in Table 3. 

### 3.3. Protein, DNA and RNA Contents in the Jejunal and Ileal Mucosa

Exposure to diquat decreased (*p* < 0.05) the jejunal mucosal protein content, as well as the ileal mucosal protein content and the ratio of RNA to DNA. In diquat-treated piglets, dietary puerarin increased (*p* < 0.05) the protein content in the jejunal mucosa, as well as the protein content, RNA to DNA ratio and protein to DNA ratio in the ileum mucosa, as displayed in Table 4.

### 3.4. PCNA Positive Cells in the Jejunum and Ileum

**Figure 1:**
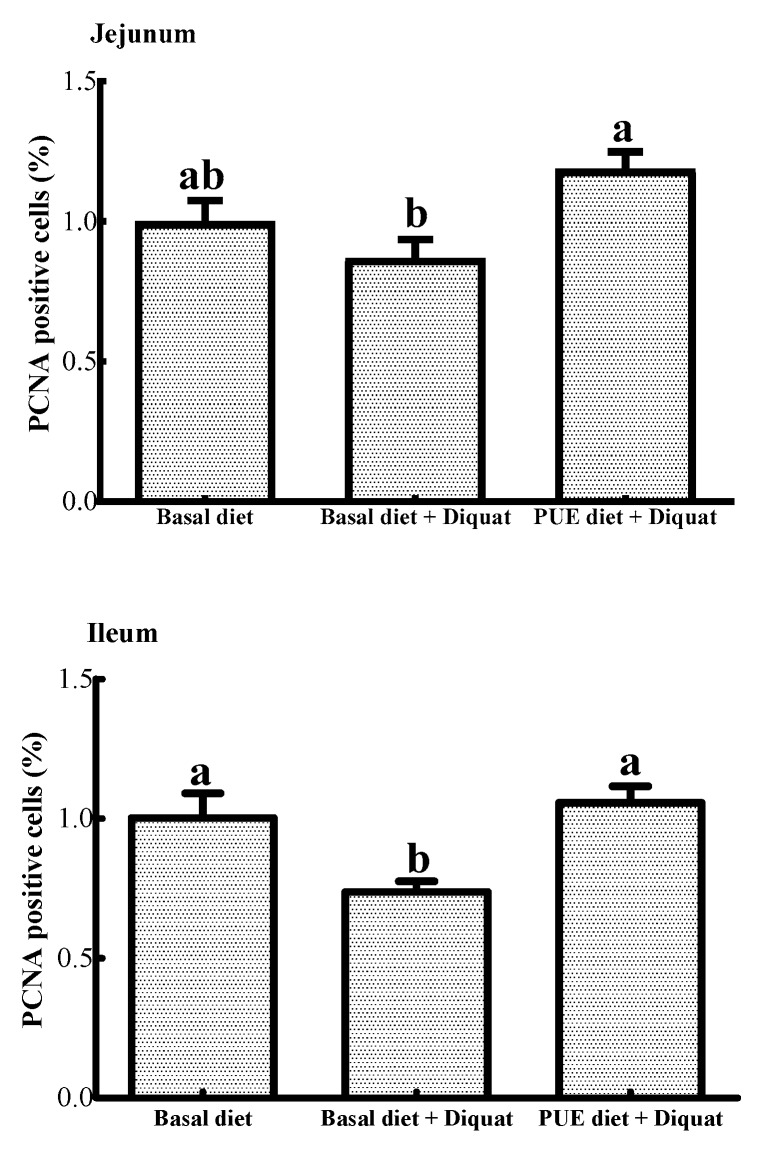
The percentage of proliferating cell nuclear antigen (PCNA) positive cells in the jejunum and ileum of the piglets. Data are expressed as means ± SEM, n =8. ^a,b^ Values with different lowercase letters are different (*p* < 0.05).

As shown in Figure 1, the percentage of PCNA positive cells in the ileum of the piglets treated with the basal diet + diquat was decreased (*p* < 0.05) when compared to that of the piglets on the basal diet treatment. The puerarin supplementation restored the inhibitory effect of the jejunal and ileal cell proliferation caused by diquat (*p* < 0.05).

### 3.5. The Relative mRNA Levels of Intercellular Junction Proteins in the Jejunum and Ileum

**Figure 2:**
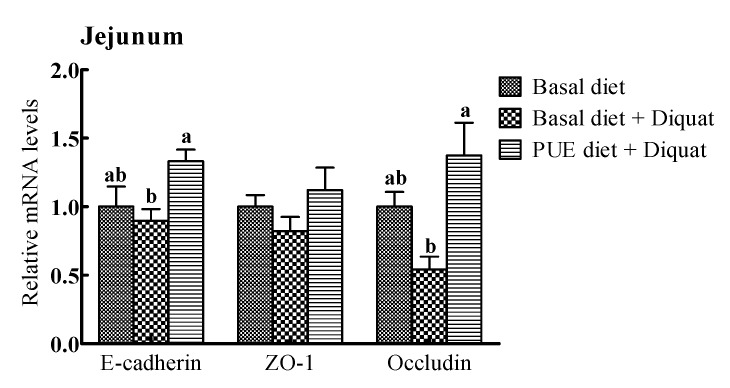

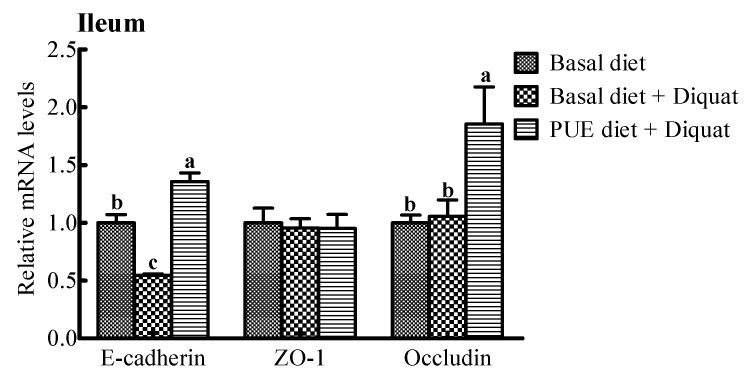
The relative mRNA levels of E-cadherin, zonula occludens (ZO-1) and occludin in the jejunum and ileum of the piglets. The mRNA expressions were expressed relative to the values of the piglets on the basal diet treatment. Data are expressed as means ± SEM, n = 8. ^a,b^ Values with different lowercase letters are different (*p* < 0.05).

The relative mRNA expressions of intercellular junction proteins (E-cadlherin, ZO-1, occludin) are shown in Figure 2. The E-cadherin and occludin mRNA expressions in the jejunum and ileum of the piglets treated with the puerarin diet were greater (*p* < 0.05) than those on the basal diet + diquat treatment. However, there were no significant differences in the ZO-1 mRNA levels in the jejunum and ileum among the piglets receiving the three treatments (*p* > 0.05). 

### 3.6. Antioxidant Parameters in the Jejunum and Ileum

As shown in Table 5, exposure to diquat decreased (*p* < 0.05) concentrations of GSH-Px in the jejunum, and GSH-Px and T-AOC in the ileum. However, the supplementation of puerarin increased (*p* < 0.05) jejunal concentrations of SOD, GSH-Px and T-AOC, as well as ileal concentrations of GSH-Px and T-AOC compared with those on the basal diet + diquat treatment. Concentrations of CAT and MDA in the jejunum and ileum showed no difference in the piglets on all three treatments (*p* > 0.05).

### 3.7. Protein Expressions of Nrf2, HO-1, NQO-1, GCLc and GCLm in the Jejunum

**Figure 3:**
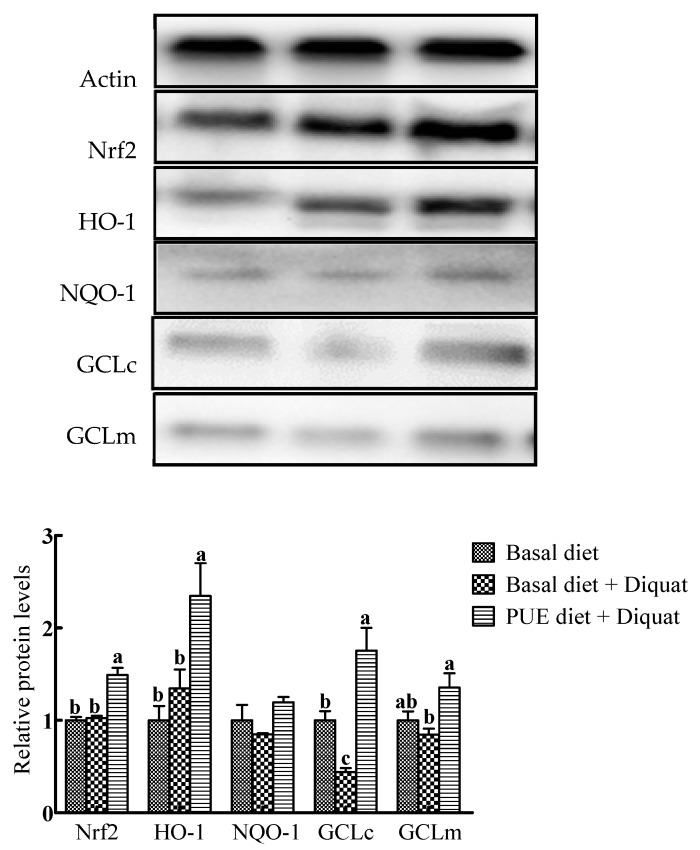
Representative Western blot images and the relative protein levels of NF-E2-related factor 2 (Nrf2), heme oxygenase-1 (HO-1), NADH dehydrogenase quinone 1(NQO-1), glutamate–cysteine ligase catalytic subunit (GCLc) and glutamate–cysteine ligase modifier subunit (GCLm) in the jejunum of piglets. The protein expressions were expressed relative to the values of the piglets on the basal diet treatment. Data are expressed as means ± SEM, n = 8. ^a-c^ Values with different lowercase letters are different (*p* < 0.05).

Representative Western blots and relative expressions of Nrf2 and antioxidant enzymes in the jejunum are shown in Figure 3. In comparison with the basal diet treatment, the protein expressions of Nrf2, HO-1 and GCLc in the piglets on the puerarin diet + diquat treatment were increased (*p* < 0.05), whereas GCLc protein expressions in the jejunum of piglets on the basal diet + diquat treatment were decreased (*p* < 0.05). The protein expressions of Nrf2, HO-1, GCLc and GCLm in the piglets on the puerarin diet + diquat treatment were greater than those on the basal diet + diquat treatment (*p* < 0.05). 

## 4. Discussion

Diquat has been widely used to induce oxidative stress, and diquat-challenging oxidative stress has been reported to affect growth performance and nutrient utilization in animals [7,16,21]. In the present study, the injection of diquat reduced growth performance and impaired gut morphology, which is in accordance with the previous report [7]. These negative effects are mainly due to the disruption in the oxidative–antioxidative balance [22]. The main antioxidant enzymes, SOD, GSH-Px and CAT, can scavenge ROS in the biological system [23,24]. The present study showed that intestinal concentrations of GSH-Px and T-AOC were decreased after exposure to diquat in the piglets on the basal diet treatment, which supports previous results in which diquat injection inhabited the activities of GSH-Px in the serum of piglets [7]. Furthermore, diquat treatment inhibited mucosal cell proliferation, and the gut barrier function showed a decreasing percentage of PCNA positive cells, as well as a relative mRNA level of E-cadherin. The present results also showed that the main antioxidant enzyme, GCLc, decreased in percentage in the jejunum after diquat injection. These results indicate that diquat induced oxidative stress in piglets, which is in accordance with the previous research [7,19,25,26].

Puerarin, the main isoflavonoid found in the Chinese herb Pueraria lobate, has been widely used in traditional Chinese medicine for thousands of years [27,28,29]. It has been reported to improve growth performance and health in animals [26,30,31]. The present study showed that there was no difference in growth performance between the piglets on the basal diet treatment and those on the puerarin diet + diquat treatment, although diquat significantly inhibited the growth of the piglets. This result indicates that the dietary supplementation of puerarin alleviated the growth performance impairment induced by diquat. 

The previous study has shown that the supplementation of Eucommia ulmoides flavones can restore the intestinal morphological structure and barrier function in piglets [7]. Puerarin has also been demonstrated to improve the structure and barrier function in cells [32]. In the present study, a higher rate of villous height to crypt depth in the jejunum indicates that dietary puerarin improved the jejunal morphological structure to some extent. E-cadherin and occludin are the main transmembrane proteins in the apical junctional complex (AJC) [33], which regulate the paracellular diffusion of ions and small molecules across epithelial barriers [34,35]. It has been reported that disorder of the AJC is a common feature of numerous inflammatory diseases [36]. For example, down-regulations of occludin and E-cadherin are linked with epithelial-mesenchymal transition [37]. The decreased expression of occludin has been considered to be associated with the staging, invasiveness and metastatic potential of epithelial cancers [37,38,39,40]. Thus, the increased expression of E-cadherin and occludin in the present study evidences that the intestinal barrier function was restored in the diquat-induced piglets treated with purarin. The improvement in the mucosal barrier function may indicate the alleviation of oxidative stress [41].

In agreement with the effect of puerarin on the intestinal barrier function, the percentage of PCNA positive cells in the jejunal and ileal mucosa increased in response to the puerarin administration. This result indicates that puerarin promoted mucosal cell proliferation, and this was possible due to the regulation of puerarin on DNA synthesis. The proliferation of eucaryon occurs in the way of mitosis. Cell division begins with intermitosis, where DNA, RNA, and protein are synthesized to prepare for the mitotic cycle [42]. The present study showed that the contents of DNA, RNA and protein in the ileum significantly increased after the administration of dietary puerarin. The increased synthesis of DNA may reflect more activities of cell proliferation. Previous studies have reported that puerarin enhanced the proliferation of human bone marrow stromal cells, and attenuated the proliferation of diabetes-induced vascular smooth muscle cells and bladder cancer cells [43,44,45], all of which is also beneficial to animals. 

In this study, significant up-regulations of SOD, GSH-Px, and T-AOC activities in the intestinal mucosa of diquat-induced piglets were observed after puerarin exposure. The up-regulation of antioxidant enzymes indicated a positive effect of puerarin on antioxidant capacity. The mechanisms of the antioxidant capacity of puerarin include the scavenging of ONOONO and total ROS, as well as the inhibition of ONOO-mediated tyrosine nitration [13]. These functions are a consequence of the activities of antioxidant enzymes such as SOD, GSH-Px, and T-AOC. SOD has been considered the first line of defense against the deleterious effects of oxyradicals on the cell, by converting superoxide radicals into hydrogen peroxide and molecular oxygen [46]. GSH-Px converts glutathione from its reduced form into its oxidative form by catalyzing hydrogen peroxide into hydrogen oxide [21,24].

GCL is the main rate-limiting enzyme for the de novo synthesis of intracellular GSH, which is one of the most versatile cellular antioxidants [47]. Interestingly, all enzymes involved in GSH biosynthesis are controlled by Nrf2 [48]. Therefore, Nrf2 plays an important role in the modulation of oxidative stress and homeostasis [49]. When stimulated by inducers, Nrf2 is released from the Kelch-like ECH-associated protein 1 (Keap1), and translocates into the nucleus where it dimerizes with cofactors and binds to ARE [50,51], thus activating downstream enzymes, including NQO1, HO-1, GCLc, and GCLm that protect cells against oxidative stress damage [51,52]. HO-1, a well-characterized cytoprotective gene controlled by Nrf2, is a microsomal enzyme that cleaves heme to produce biliverdin, carbon monoxide, and iron [53]. Mice lacking Nrf2 exhibit lower GSH levels and GCLc expression [54]. The present study showed that puerarin supplementation markedly promoted the protein expressions of Nrf2, HO-1, GCLc and GCLm in diquat-induced piglets. These results suggest that puerarin could alleviate oxidative injury involving the Nrf2 pathway caused by diquat. 

## 5. Conclusions

This study shows that puerarin has potent protective effects against diquat-induced oxidative stress. The results indicate that dietary puerarin supplementation alleviated the intestinal damage, oxidative stress, and Nrf2 pathway induced by diquat in the piglets. These findings will be helpful for the development of antibiotic-free diets, new anti-inflammatory drugs and the application of puerarin in piglets.

## Figures and Tables

**Table 1:** Composition of Basal Diets (as-fed basis).

Ingredients, %		Chemical Composition ^b^	
Corn	57	Crude protein	17.4
Expended maize	5	Digestible energy, kcal/kg	3466
Soybean meal (43%CP)	22	Lysine	0.79
Rice bran meal	5	Calcium	0.68
Broken rice	5	Total phosphorus	0.53
Fish meal	2	Dry matter	87.86
Sucrose	1		
Calcium lactate	0.3		
Calcium hydrogen phosphate	1		
Limestone powder	0.1		
Premix ^a^	1		
98% lysine	0.4		
Threonine	0.1		
Methionine	0.1		

^a^ Providing the following amounts of vitamins and minerals per kilogram on an as-fed basis: Zn (ZnO), 50 mg; Cu (CuSO4), 20 mg; Mn (MnO), 55 mg; Fe (FeSO4), 100 mg; I (KI), 1 mg; Co (CoSO4), 2 mg; Se (Na2SeO3), 0.3 mg; vitamin A, 8255 IU; vitamin D3, 2000 IU; vitamin E, 40 IU; vitamin B1, 2 mg; vitamin B2, 4 mg; pantothenic acid, 15 mg; vitamin B6, 10 mg; vitamin B12, 0.05 mg; nicotinic acid, 30 mg; folic acid, 2 mg; vitamin K3, 1.5 mg; biotin, 0.2 mg; choline chloride, 800 mg; and vitamin C, 100 mg. The premix did not contain additional copper, zinc, antibiotics, or probiotics. ^b^ Analyzed values excluding calculated digestible energy.

**Table 2:** Effects of dietary puerarin supplementation on the growth performance of the piglets.

Item	Basal Diet	Basal Diet + Diquat	Puerarin Diet + Diquat	*p*-Value
Initial BW, kg	7.26 ± 0.54	7.28 ± 0.34	7.24 ± 0.49	0.998
Final BW, kg	12.47 ± 0.67 ^a^	10.25 ± 0.39 ^b^	11.58 ± 0.63 ^ab^	0.040
ADG, g/d	372.14 ± 45.32 ^a^	212.14 ± 46.56 ^b^	310.00 ± 36.45 ^ab^	0.048
ADFI, g/d	521.36 ± 23.48 ^a^	378.21 ± 45.45 ^b^	504.32 ± 34.56 ^ab^	0.019
G:F, g/g	0.71 ± 0.04 ^a^	0.56 ± 0.05 ^b^	0.61 ± 0.02 ^ab^	0.035

Values are the mean ± SEM, n = 8 per treatment group. ^a,b^ Values with different letters within the same row are significantly different (*p* < 0.05). BW, body weight; ADG, average daily weight gain; ADFI, average daily feed intake; G:F, gain:feed.

**Table 3:** Effects of dietary puerarin supplementation on jejunal and ileal morphology in the piglets.

Item	Basal Diet	Basal Diet + Diquat	Puerarin Diet + Diquat	*p*-Value
Jejunum				
Villous height, μm	395.76 ± 7.21 ^a^	318.47 ± 21.08 ^b^	346.35 ± 27.35 ^ab^	0.042
Crypt depth, μm	106.15 ± 15.19	131.47 ± 21.49	115.34 ± 17.34	0.616
Villus height:crypt depth	3.73 ± 0.12 ^a^	2.42 ± 0.21 ^b^	3.00 ± 0.16 ^a^	<0.001
Ileum				
Villous height, μm	328.45 ± 16.79	286.42 ± 37.47	307.56 ± 35.68	0.645
Crypt depth, μm	97.14 ± 5.14	106.37 ± 8.48	101.45 ± 7.45	0.665
Villus height:crypt depth	3.38 ± 0.09 ^a^	2.69 ± 0.15 ^b^	3.03 ± 0.23 ^ab^	0.028

Values are the mean ± SEM, n = 8 per treatment group. ^a,b^ Values with different letters within the same row are significantly different (*p* < 0.05).

**Table 4:** Effects of dietary puerarin supplementation on the protein, DNA and RNA contents in the jejunal and ileal mucosa of the piglets.

Item	Basal Diet	Basal Diet + Diquat	Puerarin Diet + Diquat	*p*-Value
Jejunum				
Protein (mg/kg tissue)	67.52 ± 2.13 ^a^	56.57 ± 3.16 ^b^	64.37 ± 1.34 ^ab^	0.010
RNA/DNA	2.32 ± 0.56	2.89 ± 0.32	2.76 ± 0.25	0.580
Protein/DNA (mg/μg)	0.152 ± 0.02 ^ab^	0.134 ± 0.01 ^b^	0.187 ± 0.01 ^a^	0.045
Ileum				
Protein (mg/kg tissue)	63.23 ± 1.14 ^a^	59.25 ± 0.35 ^b^	62.96 ± 1.24 ^a^	0.017
RNA/DNA	7.15 ± 0.25 ^a^	5.15 ± 0.43 ^b^	6.46 ± 0.37 ^a^	0.003
Protein/DNA (mg/μg)	0.29 ± 0.01 ^ab^	0.24 ± 0.02 ^b^	0.30 ± 0.01 ^a^	0.022

Values are the mean ± SEM, n = 8 per treatment group. ^a,b^ Values with different letters within the same row are significantly different (*p* < 0.05).

**Table 5:** Effects of dietary puerarin supplementation on the jejunal and ileal mucosal concentrations of SOD, GSH-Px, CAT, MDA, T-AOC, GSH in the piglets.

Item	Basal Diet	Basal Diet + Diquat	Puerarin Diet + Diquat	*p*-Value
Jejunum				
SOD, U/mg prot	14.32 ± 1.06 ^ab^	9.59 ± 2.07 ^b^	15.15 ± 1.35 ^a^	0.041
GSH-Px, U/mg prot	29.35 ± 1.43 ^a^	22.13 ± 2.17 ^b^	29.87 ± 2.26 ^a^	0.020
CAT, U/mg prot	11.01 ± 0.78	9.58 ± 0.68	10.98 ± 0.79	0.326
MDA, nmol/mg prot	1.89 ± 0.18	2.36 ± 0.32	2.24 ± 0.21	0.385
T-AOC, U/mg prot	0.45 ± 0.04 ^ab^	0.32 ± 0.03b	0.54 ± 0.05 ^a^	0.003
GSH, mg/mg prot	263.34 ± 45.63	243.45 ± 35.12	276.24 ± 21.54	0.807
		Ileum		
SOD, U/mg prot	17.32 ± 1.46	16.43 ± 1.87	16.89 ± 1.21	0.920
GSH-Px, U/mg prot	24.54 ± 1.17 ^a^	18.87 ± 2.04 ^b^	25.14 ± 1.76 ^a^	0.030
CAT, U/mg prot	12.47 ± 1.34	10.42 ± 2.01	11.47 ± 0.86	0.626
MDA, nmol/mg prot	2.05 ± 0.32	2.17 ± 0.47	1.94 ± 0.15	0.892
T-AOC, U/mg prot	0.53 ± 0.02 ^a^	0.37 ± 0.04 ^b^	0.59 ± 0.06 ^a^	0.006
GSH, mg/mg prot	246.46 ± 40.21	216.75 ± 31.06	218.49 ± 29.97	0.789

Values are the mean ± SEM, n = 8 per treatment group. ^a,b^ Values with different letters within the same row are significantly different (*p* < 0.05). SOD, superoxide dismutase; GSH-Px, glutathione peroxidase; CAT, catalase; MDA, malondialdehyde; T-AOC, total antioxidant capacity; GSH, glutathione.

## Data Availability

All data generated or analyzed during this study are available from the corresponding author on reasonable request.
